# Chronic Cough and Speech Therapy

**DOI:** 10.1590/2317-1782/20212021127

**Published:** 2021-10-29

**Authors:** Rodrigo Dornelas, Vanessa Veis Ribeiro, Mara Behlau

**Affiliations:** 1 Universidade Federal de São Paulo – UNIFESP - São Paulo (SP), Brasil.; 2 Universidade Federal do Rio de Janeiro – UFRJ - Rio de Janeiro (RJ), Brasil.; 3 Universidade Federal de Sergipe – UFS - Lagarto (SE), Brasil.; 4 Centro de Estudos da Voz – CEV - São Paulo (SP), Brasil.

Dear chief-editors

Ana Luiza Gomes Pinto Navas, Anna Alice Figueiredo de Almeida and Stela Maris Aguiar Lemos,

The professional of speech-language therapist is about to celebrate 40 years of regulation in Brazil and is now looking into a new line of work: chronic cough, which is already recognized in other countries, such as Australia^(1,2)^ and United States of America^(3)^.

Traditional studies in speech therapy addressed the larynx according to two areas regarding its function: voice as a phonatory function, and dysphagia as a protective function for the lower airways. Speech-language therapists now face a new challenge: to contribute to the assessment and treatment of patients with chronic refractory cough, an area that involves specific expertise, in addition to, as any other field, requiring professional training and preparation. In cases of refractory chronic cough, the larynx is the main focus for allowing an overlap between the specialties of voice and dysphagia, towards a direct interest in the assessment and treatment of these patients. Individuals with chronic cough may or may not have dysphonia and/or dysphagia; therefore, some cases can become more complex, thus requiring better structured clinical reasoning by using the combined resources of a hybrid approach relying on elements of the hypothetical-deductive model and the theory of construction of disease scripts, applied to speech-language therapy intervention^(4)^.

The first mention of chronic cough as psychogenic cough in the literature, or at least the first known at the time appeared in the early 1980s ^(5)^. Soon after, interest in the area begins to arise through publications on assessment and intervention strategies in cases of patients with chronic cough^(1,6,7)^. Evidence on the effects of speech-language therapy interventions begins to emerge and the rehabilitation of patients with cough by speech-language therapists starts to be recognized outside the profession, being indicated by multidisciplinary teams^(1)^.

Cough is a protective mechanism resulting from a complex reflex initiated by the activation of irritating receptors in the airway^(8)^, constituting a forced expulsion maneuver, usually against a closed glottis^(9)^.

When lasting up to three weeks, it is considered acute and beneficial to the respiratory system through the removal of harmful substances and increased mucociliary clearance; however, chronic cough has no benefit for the respiratory system or the body in general^(8)^. When persisting for more than eight weeks, cough becomes chronic. An exception to the rule is in cases of upper respiratory tract infections, in which coughing for up to eight weeks is considered acceptable^(10)^. To be categorized as refractory, cough needs to be persistent under medical treatment for specific causes, such as respiratory diseases and gastroesophageal reflux^(11)^. Chronic refractory cough is a difficult problem often associated with increased cough reflex sensitivity^(7)^, occurring in up to 46% of patients with chronic cough^(2,12)^. The main features of refractory chronic cough are abnormal sore throat or tickling sensation (laryngeal paresthesia), greater cough sensitivity in response to coughing agents (hypertension), and cough triggered by non-cough stimuli, such as talking or cold air (allotussia)^(13)^ *.* In addition, up to 40% of people with this condition suffer from vocal problems, while about 56% may also have paradoxical vocal fold movement^(2)^.

Speech-language therapy assessment is based on clinical procedures (patient’s clinical examination, cough frequency and threshold, auditory-perceptual, acoustic, and aerodynamic evaluation of the voice), in addition to self-assessment instruments^(3,7,14,15)^. The following questionnaires are already validated in Brazilian Portuguese: Cough Severity Index (CSI-Br), which measures self-perceived severity of cough symptoms^(16)^, and Newcastle Laryngeal Hypersensitivity Questionnaire (LHQ-Br), which measures self-perception of laryngeal sensations associated with laryngeal hypersensitivity syndrome^(17)^. In turn, the following protocols are translated and adapted into Brazilian Portuguese: Vocal Disadvantage Index – Throat (VDI-G)(18), to measure the perceived vocal handicap related to the symptoms of throat, and Leicester Questionnaire^(19)^, to assess cough symptom and its impact on the health status of patients with chronic cough. Both protocols are already in the process of being validated for Brazilian Portuguese with the definition of their psychometric properties.

Speech-language rehabilitation has been shown to be a potentially efficient intervention in the management of chronic refractory cough by seeking to break the cycle of irritation in cough recipients. Failure of medical intervention ^(8)^ may or may not be associated with the use of anti-cough drugs^(20 ^–^ 22)^. Treatment approaches for chronic refractory cough include active cough suppression, reduced cough reflex sensitivity, or higher cough reflex threshold^(7,23)^, in addition to reduced laryngeal irritation^(7)^.

More recently, a proposal has been elaborated for speech-language therapy rehabilitation in Brazil, called Therapy Program for the Management of Chronic Cough (TMTC), aimed at treating chronic refractory cough^(24)^. The international literature introduces programs of different nature as well, such as physiotherapy, speech and language therapy intervention (PSALTI)^(25)^, and the SPEech Pathology Intervention Program for CHronic Cough (SPEICH-C)^(26)^.

This scenario shows that Brazilian speech-language therapy is concerned with obtaining validation of instruments and producing evidence to explore such important and promising area, seeking to contribute to improve patients’ life quality in the scope of chronic refractory cough. Speech-language therapists who are specialists in voice and dysphagia are potentially responsible for contributing to the diagnosis and treatment of this important laryngeal dysfunction.
