# Erratum: chronic coughand speech therapy

**DOI:** 10.1590/2317-1782/20212021307

**Published:** 2022-02-21

Rodrigo Dornelas^1,2^, Vanessa Veis Ribeiro^1,3^, Mara Behlau^1,4^

^1^Universidade Federal de São Paulo – UNIFESP – São Paulo (SP), Brasil.

^2^Universidade Federal do Rio de Janeiro – UFRJ – Rio de Janeiro (RJ), Brasil.

^3^Universidade Federal de Sergipe – UFS – Lagarto (SE), Brasil.

^4^Centro de Estudos da Voz – CEV – São Paulo (SP), Brasil.

Due to technical problems during the editorial production the article “Chronic Coughand Speech Therapy”, DOI https://doi.org/10.1590/2317-1782/20212021127, published in CoDAS, *34(1)*, e20210127, 2022, was published with the error of losing random spaces in parts of the text.

**On English title of the Portuguese and English versions, where the text reads:**

Chronic Coughand Speech Therapy

**It should read:**

Chronic Cough and Speech Therapy

**On correspondence address of the English version, where the text reads:**

RuaBotucatu, 802, Vila

Clementino, São Paulo (SP), Brasil,

CEP: 04023-062

**It should read:**

Rua Botucatu, 802, Vila

Clementino, São Paulo (SP), Brasil,

CEP: 04023-062

**On references of the Portuguese and English versions, where the text reads:**

1. GibsonPG, VertiganAE. Speech pathology for chroniccough: a new approach.PulmPharmacolTher. 2009;22(2):159-62. http://dx.doi.org/10.1016/j.pupt.2008.11.005. PMid:19061964.

2. RyanNM, GibsonPG. Recentadditions in thetreatmentofcough.J ThoracDis. 2014;6(suppl. 7):S739-47. PMid:25383209.

3. CarrollTL. Chroniccough.San Diego, CA: LOGO Plural Publishing; 2019. 220 p.

4. PeixotoJM, SantosSME, FariaRMD. Clinicalreasoningdevelopment in medical students.RevBrasEduc Med. 2018;42(1):73-81.

5. BlagerFB, GayML, WoodRP. Voice therapytechniquesadaptedtotreatmentofhabitcough: a pilotstudy.J CommunDisord. 1988;21(5):393-400. http://dx.doi.org/10.1016/0021-9924(88)90024-X. PMid:3183084.

6. VertiganAE, TheodorosDG, GibsonPG, WinkworthAL. Efficacyof speech pathology management for chroniccough: a randomised placebo controlledtrialoftreatmentefficacy.Thorax. 2006;61(12):1065-9. http://dx.doi.org/10.1136/thx.2006.064337. PMid:16844725.

7. RyanNM, VertiganAE, BoneS, GibsonPG. Coughreflexsensitivity improves with speech languagepathology management ofrefractorychroniccough.Cough. 2010;6(1):5. http://dx.doi.org/10.1186/1745-9974-6-5. PMid:20663225.

8. VertiganAE, TheodorosDG, GibsonPG, WinkworthAL. The relationshipbetweenchroniccoughandparadoxical vocal foldmovement: a review oftheliterature.J Voice. 2006;20(3):466-80. http://dx.doi.org/10.1016/j.jvoice.2005.08.001. PMid:16274959.

9. MoriceAH, McGarveyL, PavordI. Recommendations for the management ofcough in adults.Thorax. 2006;61(suppl. 1):i1-24. http://dx.doi.org/10.1136/thx.2006.065144. PMid:16936230.

10. PouloseV, MohdIB. Prolongedcoughpresentingwithdiagnosticdifficulty: a studyofaetiologicalandclinicaloutcomes.Singapore Med J. 2011;52(4):267-70. PMid:21552788.

11. ChungKF, PavordID. Prevalence, pathogenesis, and causes ofchroniccough.Lancet. 2008;371(9621):1364-74. http://dx.doi.org/10.1016/S0140-6736(08)60595-4. PMid:18424325.

12. VertiganAE, KapelaSM, FrankeI, GibsonPG. The effectof a vocal loadingtestoncoughandphonation in patientswithchroniccough.J Voice. 2017;31(6):763-72. http://dx.doi.org/10.1016/j.jvoice.2017.03.020. PMid:28461166.

13. RyanNM, BirringSS, GibsonPG. Gabapentin for refractorychroniccough: a randomised, double-blind, placebo-controlledtrial.Lancet. 2012;380(9853):1583-9. http://dx.doi.org/10.1016/S0140-6736(12)60776-4. PMid:22951084.

14. VertiganAE, BoneSL, GibsonPG. Developmentandvalidationofthe Newcastle laryngealhypersensitivityquestionnaire.Cough. 2014;10(1):1. http://dx.doi.org/10.1186/1745-9974-10-1. PMid:24552215.

15. VertiganA, GibsonP. Speech Pathology management ofCronicRefractoryCoughandrelateddisorders.Oxford, UK: CoptomPublishing; 2016.

16. RibeiroVV, LopesLW, SilvaACF, MedeirosAHNo, Gartner-SchmidtJ, BehlauM. Coughseverity index: validation in BrazilianPortuguese. J Voice. [Internet]. 2021Jul [citado em 2021 Maio 5]. Disponível em: https://www.sciencedirect.com/science/article/abs/pii/S0892199721002022

17. RibeiroVV, LopesLW, SilvaACF, MedeirosAHNo, VertiganA, BehlauM. Validationof Newcastle LaryngealHypersensitivityQuestionnaire (LHQ-Br) in BrazilianPortuguese. J Voice. [Internet]. 2021Jul [citado em 2021 Maio 5]. Disponível em: https://www.sciencedirect.com/science/article/abs/pii/S0892199721002009

18. RibeiroVV, LopesLW, SilvaACF, MedeirosAHNo, Lyberg-ÅhlanderV, SchalenL, et al. Voice handicap index-throat: translationandcross-cultural adaptationtoBrazilianPortuguese. J Voice. [Internet]. 2020Maio [citado em 2021 Maio 5]. Disponível em: https://linkinghub.elsevier.com/retrieve/pii/S0892199720301338

19. FelisbinoMB, SteidleLJM, Gonçalves-TavaresM, PizzichiniMMM, PizzichiniE. Leicester CoughQuestionnaire: translationtoPortugueseandcross-cultural adaptation for use in Brazil.J BrasPneumol. 2014;40(3):213-21. http://dx.doi.org/10.1590/S1806-37132014000300003. PMid:25029643.

20. VertiganAE, KapelaSL, RyanNM, BirringSS, McElduffP, GibsonPG. Pregabalinand speech pathologycombinationtherapy for refractorychroniccough a randomizedcontrolledtrial.Chest. 2016;149(3):639-48. http://dx.doi.org/10.1378/chest.15-1271. PMid:26447687.

21. GibsonP, WangG, McGarveyL, VertiganAE, AltmanKW, BirringSS, et al. Treatmentofunexplainedchroniccoughchestguidelineand expert panel report.Chest. 2016;149(1):27-44. http://dx.doi.org/10.1378/chest.15-1496. PMid:26426314.

22. ChamberlainS, BirringSS, GarrodR. Nonpharmacologicalinterventions for refractorychroniccoughpatients: systematic review.Lung. 2014;192(1):75-85. http://dx.doi.org/10.1007/s00408-013-9508-y. PMid:24121952.

23. ChamberlainS, GarrodR, BirringSS. Coughsuppressiontherapy: does it work?PulmPharmacolTher. 2013;26(5):524-7. http://dx.doi.org/10.1016/j.pupt.2013.03.012. PMid:23524013.

24. RibeiroVV, LopesLW, BehlauM. PresentationoftheTherapyProgram for Management ofChronicCough.CoDAS. 2021;33(3):e20200057. http://dx.doi.org/10.1590/2317-1782/20202020057. PMid:34076101.

25. MitchellSAFC, GarrodR, ClarkL, DouiriA, ParkerSM, EllisJ, et al. Physiotherapy, and speech andlanguagetherapyintervention for patientswithrefractorychroniccough: a multicentrerandomisedcontroltrial.Thorax. 2017;72(2):129-36. http://dx.doi.org/10.1136/thoraxjnl-2016-208843. PMid:27682331.

26. VertiganAE, TheodorosDG, WinkworthAL, GibsonPG. A comparisonoftwo approaches tothetreatmentofchroniccough: perceptual, acoustic, andelectroglottographicoutcomes.J Voice. 2008;22(5):581-9. http://dx.doi.org/10.1016/j.jvoice.2007.01.001. PMid:17485195.

It should read:

1. Gibson PG, Vertigan AE. Speech pathology for chronic cough: a new approach. Pulm Pharmacol Ther. 2009;22(2):159-62. http://dx.doi.org/10.1016/j.pupt.2008.11.005. PMid:19061964.

2. Ryan NM, Gibson PG. Recent additions in the treatment of cough. J Thorac Dis. 2014;6(suppl. 7):S739-47. PMid:25383209.

3. Carroll TL. Chronic cough. San Diego, CA: LOGO Plural Publishing; 2019. 220 p.

4. Peixoto JM, Santos SME, Faria RMD. Clinical reasoning development in medical students. Rev Bras Educ Med. 2018;42(1):73-81.

5. Blager FB, Gay ML, Wood RP. Voice therapy techniques adapted to treatment of habit cough: a pilot study. J Commun Disord. 1988;21(5):393-400. http://dx.doi.org/10.1016/0021-9924(88)90024-X. PMid:3183084.

6. Vertigan AE, Theodoros DG, Gibson PG, Winkworth AL. Efficacy of speech pathology management for chronic cough: a randomised placebo controlled trial of treatment efficacy. Thorax. 2006;61(12):1065-9. http://dx.doi.org/10.1136/thx.2006.064337. PMid:16844725.

7. Ryan NM, Vertigan AE, Bone S, Gibson PG. Cough reflex sensitivity improves with speech language pathology management of refractory chronic cough. Cough. 2010;6(1):5. http://dx.doi.org/10.1186/1745-9974-6-5. PMid:20663225.

8. Vertigan AE, Theodoros DG, Gibson PG, Winkworth AL. The relationship between chronic cough and paradoxical vocal fold movement: a review of the literature. J Voice. 2006;20(3):466-80. http://dx.doi.org/10.1016/j.jvoice.2005.08.001. PMid:16274959.

9. Morice AH, McGarvey L, Pavord I. Recommendations for the management of cough in adults. Thorax. 2006;61(suppl. 1):i1-24. http://dx.doi.org/10.1136/thx.2006.065144. PMid:16936230.

10. Poulose V, Mohd IB. Prolonged cough presenting with diagnostic difficulty: a study of aetiological and clinical outcomes. Singapore Med J. 2011;52(4):267-70. PMid:21552788.

11. Chung KF, Pavord ID. Prevalence, pathogenesis, and causes of chronic cough. Lancet. 2008;371(9621):1364-74. http://dx.doi.org/10.1016/S0140-6736(08)60595-4. PMid:18424325.

12. Vertigan AE, Kapela SM, Franke I, Gibson PG. The effect of a vocal loading test on cough and phonation in patients with chronic cough. J Voice. 2017;31(6):763-72. http://dx.doi.org/10.1016/j.jvoice.2017.03.020. PMid:28461166.

13. Ryan NM, Birring SS, Gibson PG. Gabapentin for refractory chronic cough: a randomised, double-blind, placebo-controlled trial. Lancet. 2012;380(9853):1583-9. http://dx.doi.org/10.1016/S0140-6736(12)60776-4. PMid:22951084.

14. Vertigan AE, Bone SL, Gibson PG. Development and validation of the Newcastle laryngeal hypersensitivity questionnaire. Cough. 2014;10(1):1. http://dx.doi.org/10.1186/1745-9974-10-1. PMid:24552215.

15. Vertigan A, Gibson P. Speech Pathology management of Cronic Refractory Cough and related disorders. Oxford, UK: Coptom Publishing; 2016.

16. Ribeiro VV, Lopes LW, Silva ACF, Medeiros AH No, Gartner-Schmidt J, Behlau M. Cough severity index: validation in Brazilian Portuguese. J Voice. [Internet]. 2021 Jul [citado em 2021 Maio 5]. Disponível em: https://www.sciencedirect.com/science/article/abs/pii/S0892199721002022

17. Ribeiro VV, Lopes LW, Silva ACF, Medeiros AH No, Vertigan A, Behlau M. Validation of Newcastle Laryngeal Hypersensitivity Questionnaire (LHQ-Br) in Brazilian Portuguese. J Voice. [Internet]. 2021 Jul [citado em 2021 Maio 5]. Disponível em: https://www.sciencedirect.com/science/article/abs/pii/S0892199721002009

18. Ribeiro VV, Lopes LW, Silva ACF, Medeiros AH No, Lyberg-Åhlander V, Schalen L, et al. Voice handicap index-throat: translation and cross-cultural adaptation to Brazilian Portuguese. J Voice. [Internet]. 2020 Maio [citado em 2021 Maio 5]. Disponível em: https://linkinghub.elsevier.com/retrieve/pii/S0892199720301338

19. Felisbino MB, Steidle LJM, Gonçalves-Tavares M, Pizzichini MMM, Pizzichini E. Leicester Cough Questionnaire: translation to Portuguese and cross-cultural adaptation for use in Brazil. J Bras Pneumol. 2014;40(3):213-21. http://dx.doi.org/10.1590/S1806-37132014000300003. PMid:25029643.

20. Vertigan AE, Kapela SL, Ryan NM, Birring SS, McElduff P, Gibson PG. Pregabalin and speech pathology combination therapy for refractory chronic cough a randomized controlled trial. Chest. 2016;149(3):639-48. http://dx.doi.org/10.1378/chest.15-1271. PMid:26447687.

21. Gibson P, Wang G, McGarvey L, Vertigan AE, Altman KW, Birring SS, et al. Treatment of unexplained chronic cough chest guideline and expert panel report. Chest. 2016;149(1):27-44. http://dx.doi.org/10.1378/chest.15-1496. PMid:26426314.

22. Chamberlain S, Birring SS, Garrod R. Nonpharmacological interventions for refractory chronic cough patients: systematic review. Lung. 2014;192(1):75-85. http://dx.doi.org/10.1007/s00408-013-9508-y. PMid:24121952.

23. Chamberlain S, Garrod R, Birring SS. Cough suppression therapy: does it work? Pulm Pharmacol Ther. 2013;26(5):524-7. http://dx.doi.org/10.1016/j.pupt.2013.03.012. PMid:23524013.

24. Ribeiro VV, Lopes LW, Behlau M. Presentation of the Therapy Program for Management of Chronic Cough. CoDAS. 2021;33(3):e20200057. http://dx.doi.org/10.1590/2317-1782/20202020057. PMid:34076101.

25. Mitchell SAFC, Garrod R, Clark L, Douiri A, Parker SM, Ellis J, et al. Physiotherapy, and speech and language therapy intervention for patients with refractory chronic cough: a multicentre randomised control trial. Thorax. 2017;72(2):129-36. http://dx.doi.org/10.1136/thoraxjnl-2016-208843. PMid:27682331.

26. Vertigan AE, Theodoros DG, Winkworth AL, Gibson PG. A comparison of two approaches to the treatment of chronic cough: perceptual, acoustic, and electroglottographic outcomes. J Voice. 2008;22(5):581-9. http://dx.doi.org/10.1016/j.jvoice.2007.01.001. PMid:17485195.

We apologize for the errors.

