# Supplementary material for: Chronic Cough and Speech Therapy
Source: Codas. 2021 Oct 29;34(1):e20210127. doi: 10.1590/2317-1782/20212021127 (PMC9886112; doi:10.1590/2317-1782/20212021127)
Supplement: Translation

# Translation: Tosse Crônica e Fonoaudiologia

**Authors:** Rodrigo Dornelas, Vanessa Veis Ribeiro, Mara Behlau

**Affiliations:** 1 Universidade Federal de São Paulo – UNIFESP - São Paulo (SP), Brasil.; 2 Universidade Federal do Rio de Janeiro – UFRJ - Rio de Janeiro (RJ), Brasil.; 3 Universidade Federal de Sergipe – UFS - Lagarto (SE), Brasil.; 4 Centro de Estudos da Voz – CEV - São Paulo (SP), Brasil.

Prezadas editoras-chefes

Ana Luiza Gomes Pinto Navas, Anna Alice Figueiredo de Almeida e Stela Maris Aguiar Lemos,

O fonoaudiólogo brasileiro, prestes a comemorar 40 anos de regulamentação da profissão, debruça-se em um novo seguimento de atuação: a tosse crônica, o que já é reconhecido em outros países, como Austrália^(1,2)^ e Estados Unidos^(3)^.

Na Fonoaudiologia, a laringe tradicionalmente era estudada por duas áreas com atuação que envolvem sua função, a voz, enquanto função fonatória, e a disfagia, enquanto função de proteção de vias aéreas inferiores. O novo desafio para o fonoaudiólogo é contribuir na avaliação e no tratamento dos pacientes com tosse crônica refratária, uma área que envolve uma *expertise* específica e que, como todas as outras áreas, requer estudos e preparo por parte do profissional. Nos casos de tosse crônica refratária, percebe-se que o foco principal é a laringe, o que permite uma sobreposição entre as especialidades voz e disfagia, com interesse direto na avaliação e tratamento desses pacientes. Indivíduos com tosse crônica podem ou não ter disfonia e/ou disfagia e, desta forma, os casos podem adquirir grande complexidade e exigir um raciocínio clínico mais estruturado, usando recursos combinados de uma abordagem híbrida que conta com elementos do modelo hipotético-dedutivo e da teoria da construção dos *scripts* das doenças, aplicados à intervenção fonoaudiológica^(4)^.

Na literatura, a primeira citação de tosse crônica ou conhecida na época também como tosse psicogênica foi realizada no início da década de 1980 ^(5)^. Logo em seguida, nota-se um interesse nessa área, com artigos publicados sobre as estratégias de avaliação e intervenção nos casos de pacientes com tosse crônica^(1,6,7)^. As evidências sobre os efeitos da intervenção fonoaudiológica começam a surgir e a reabilitação de pacientes com tosse, por fonoaudiólogos começa a ser reconhecida fora de nossa profissão, indicada por equipes multiprofissionais^(1)^.

A tosse é um mecanismo protetor resultante de um reflexo complexo, iniciado pela ativação de receptores irritantes na via aérea^(8)^. Trata-se de uma manobra expulsiva forçada, geralmente contra uma glote fechada^(9)^.

Quando sua duração é de até três semanas ela é considerada aguda e benéfica para o sistema respiratório por meio da remoção de substâncias nocivas e do aumento da depuração mucocilar, porém, a tosse crônica não tem benefício para o sistema respiratório ou para o corpo em geral^(8)^. Ao persistir por um período superior a oito semanas, a tosse passa a ser crônica. Uma exceção à regra ocorre nos casos de infecções do trato respiratório superior, em que a tosse por até oito semanas é considerada aceitável^(10)^. Para ser denominada de refratária, a tosse precisa ser persistente ao tratamento médico para causas específicas como doenças respiratórias e refluxo gastroesofágico^(11)^. A tosse crônica refratária é um problema difícil frequentemente associado ao aumento da sensibilidade do reflexo da tosse^(7)^, e ocorre em até 46% dos pacientes com tosse crônica^(2,12)^. As principais características da tosse crônica refratária são sensação anormal de garganta ou cócegas (parestesia laríngea), aumento da sensibilidade à tosse em resposta a agentes tussígenos (hipertensão) e tosse desencadeada por estímulos não tussígenos, como falar ou ar frio *(alotussia)* ^(13)^ *.* Além disso, até 40% das pessoas com esse quadro sofrem problemas vocais e cerca de 56% também podem apresentar movimento paradoxal das pregas vocais^(2)^.

A avaliação fonoaudiológica dá-se por meio de procedimentos clínicos (exame clínico do paciente; frequência e limiar da tosse; avaliação perceptivo-auditiva, acústica e aerodinâmica da voz), além de instrumentos de autoavaliação^(3,7,14,15)^. Os questionários já validados em português brasileiro são o Índice de Severidade da Tosse (CSI-Br) que mensura a autopercepção da severidade dos sintomas de tosse^(16)^, e o Questionário Newcastle de Hipersensibilidade Laríngea (LHQ-Br) que mensura a autopercepção de sensações laríngeas associadas com a síndrome da hipersensibilidade laríngea^(17)^. Já os protocolos traduzidos e adaptados para o Português Brasileiro são o Índice de Desvantagem Vocal - Garganta (IDV-G)^(18)^ que mensura a desvantagem vocal percebida relacionada aos sintomas de garganta e o Questionário de Leicester^(19)^ que avalia o sintoma de tosse e seu impacto no estado de saúde dos portadores de tosse crônica, sendo que ambos já encontram-se em processo de validação para o português brasileiro, com definição de suas propriedades psicométricas.

A reabilitação fonoaudiológica tem-se mostrado uma intervenção potencialmente eficiente no manejo da tosse crônica refratária e busca quebrar o ciclo de irritação dos receptores de tosse, quando a intervenção médica falha^(8)^, podendo ou não ser associada ao uso de fármacos anti-tussígenos^(20 ^–^ 22)^. As abordagens de tratamento para a tosse crônica refratária incluem supressão ativa da tosse, redução da sensibilidade ao reflexo da tosse ou aumento do limiar do reflexo da tosse^(7,23)^, além de redução da irritação laríngea^(7)^.

Recentemente foi elaborada uma proposta de reabilitação fonoaudiológica brasileira denominada Programa de Terapia para Manejo da Tosse Crônica (TMTC) para tratamento da tosse crônica refratária^(24)^. A literatura internacional apresenta outros programas como o physiotherapy, and speech and language therapy intervention (PSALTI)^(25)^ e o SPEech Pathology Intervention Program for CHronic Cough (SPEICH-C)^(26)^.

Observa-se assim que a Fonoaudiologia brasileira mostra-se preocupada em obter validação de instrumentos e produzir evidências para explorar essa importante e promissora área de atuação, a fim de contribuir para a melhora da qualidade de vida de pacientes com tosse crônica refratária. Fonoaudiólogos especialistas em voz e em disfagia tem potencialmente a responsabilidade de contribuir no diagnóstico e tratamento dessa importante disfunção laríngea.
